# Supplementary material for: FPLV: Enhancing recommender systems with fuzzy preference, vector similarity, and user community for rating prediction
Source: PLoS One. 2023 Aug 28;18(8):e0290622. doi: 10.1371/journal.pone.0290622 (PMC10461840; doi:10.1371/journal.pone.0290622)
Supplement: Attachment — Submitted filename: 1.Response-to-Reviewers2.docx [file pone.0290622.s001.docx]

**Original Manuscript ID:** PONE-D-22-28030

**Original Article Title:** “Link prediction in recommender systems based on fuzzy preference vectors and community characteristics”

**To:** Editors

**Re:** Response to reviewers

Dear Editor,

Thank you for allowing a resubmission of our manuscript, with an opportunity to address the reviewers’ comments.

We are uploading (a) our point-by-point response to the comments (below) (response to reviewers), (b) an updated manuscript with yellow highlighting indicating changes, and (c) a clean updated manuscript without highlights (PDF main document).

Best regards,

Zhan Su, Haochuan Yang, and Jun Ai.

**All experiment data can be found at:**

**https://github.com/PlayerAI/PONE-D-22-28030**

**Reviewer: 1**

**Concern # 1:** The paper should be corrected by a native speaker to improve the English.

**Author response:** We appreciate the valuable feedback provided by the reviewer.

**Author action:** In response to the reviewer's suggestion, we have carefully reviewed and corrected the English language in our paper for further consideration. All big changes marked yellow, proofreading doesn’t marked.

**Concern # 2:** The following very recent work can be also included in the bibliography:
[1] Su, Z., Zheng, X., Ai, J., Shen, Y., & Zhang, X. (2020). Link prediction in recommender systems based on vector similarity. Physica A: Statistical Mechanics and its Applications, 560, 125154.

**Author response:** Thank you for the advice, the suggested work is actually one of our recent publications. We have added in the revised paper as advised.

**Author action:** On page 14, **w**e have added the suggested algorithm to the revised manuscript and also added another paper on recent publication by our group (labeled as SRA in experiments). We thank the reviewers for being interested in our research.
[2] Ai, J., Cai, Y., Su, Z., Zhang, K., Peng, D., & Chen, Q. (2022). Predicting user-item links in recommender systems based on similarity-network resource allocation. *Chaos, Solitons & Fractals*, *158*, 112032.

**Concern # 3:** In experimental results, you may check again the results of DTEC-SCoR algorithm that seems to yield very low values.
However, this method is a state of the art RS method that should perform better results,
so the authors have to check again their implementation and experiments.

**Author response:** Thank you for the comment, we have found the bug in our implementation and updated the corresponding results. DTEC-SCoR does very good in most of the experiments.

**Author action:** We have rewritten part of DTEC-SCoR implementation, the results is shown in the experiment sections on page 18-23.

**Concern # 4:** Please, include comparisons with method [1] that also report results on Netflix dataset.
[1] Su, Z., Zheng, X., Ai, J., Shen, Y., & Zhang, X. (2020). Link prediction in recommender systems based on vector similarity. Physica A: Statistical Mechanics and its Applications, 560, 125154.

**Author response:** Thank you for this comment. The method has been added as the reviewer suggested.

**Author action:** The updated results can be seen from pages 14 in the revised manuscript. We also replace the older Bhattacharyya CF (2015) with a newer Method SRA (2023).

**Concern # 5:** Computational complexity comparisons are missing. The values of comp. times reported on Fig. 12 are too low low concerning the size of the datasets. Please, explain better the experiment and provide the used hardware.

**Author response:** We appreciate the reviewer’s comment and have updated the manuscript accordingly.

**Author action:** We have added a paragraph to address this issue, which can be found on pages 22-23.

**Concern # 6:** Please provide some cases that the proposed method fails and explain the reasons.

**Author response:** Thanks to your suggestion, in the discussion of the algorithm and in the conclusion vignette, we have added a related discussion.

**Author action:** Corresponding revisions can be seen on pages 23 of the revised paper, where the revisions have been marked yellow. “However,…”

**Concern # 7:** minor changes
Equ. -> Eq.
Fig -> Fig.

**Author response:** Thank you for the comment.

**Author action:** The relevant abbreviations have been revised. However, the “MANUSCRIPT BODY FORMATTING GUIDELINES” of PLOS ONE suggests that Figures should be cited as “Fig 1” and equation citation should be “Eq 1”. Therefore, we keep them as the journal requires.

**Reviewer: 2**

**Concern # 1:** The paper is good written and well arranged. Author have to follow the guideline that how to arrange the text in paper.
Author can improve the Proposed Method part of the paper. Remove grammatical mistakes.

**Author response:** Thanks to the reviewer's encouragement, we revised the paper according to the journal's guideline. It was also proofread for grammar and expression.

**Author action:** The paper has been revised accordingly, all big changes marked yellow, proofreading doesn’t marked.
